# MOCA: a systematic toolbox for designing and assessing modular functional near-infrared brain imaging probes

**DOI:** 10.1117/1.NPh.9.1.017801

**Published:** 2022-02-08

**Authors:** Morris Vanegas, Miguel Mireles, Qianqian Fang

**Affiliations:** Northeastern University, Department of Bioengineering, Boston, Massachusetts, United States

**Keywords:** functional near-infrared spectroscopy, modular probe, brain sensitivity, analysis, layout, montage

## Abstract

**Significance:**

The expansion of functional near-infrared spectroscopy (fNIRS) systems toward broader utilities has led to the emergence of modular fNIRS systems composed of repeating optical source/detector modules. Compared to conventional fNIRS systems, modular fNIRS systems are more compact and flexible, making wearable and long-term monitoring possible. However, the large number of design parameters makes understanding their impact on a probe’s performance a daunting task.

**Aim:**

We aim to create a systematic software platform to facilitate the design, characterization, and comparison of modular fNIRS probes.

**Approach:**

Our software—modular optode configuration analyzer (MOCA)—implements semi-automatic algorithms that assist in tessellating user-specified regions-of-interest, in interconnecting modules of various shapes, and in quantitatively comparing probe performance using metrics, such as spatial channel distributions and average brain sensitivity of the resulting probes. There is also support for limited parameter sweeping capabilities.

**Results:**

Through several examples, we show that users can use MOCA to design and optimize modular fNIRS probes, study trade-offs between several module shapes, improve brain sensitivity in probes via module re-orientation, and enhance probe performance via adjusting module spatial layouts.

**Conclusion:**

Despite its simplicity, our modular probe design platform offers a framework to describe and quantitatively assess probes made by modules, opening a new door for the growing fNIRS user community to approach the challenging problem of module- and probe-parameter selection and fine-tuning.

## 1 Introduction

Functional near-infrared spectroscopy (fNIRS) is an emerging neuroimaging technique to non-invasively measure brain activity using non-ionizing light.^1^ Unlike functional magnetic resonance imaging (fMRI)^2^ that requires high-strength magnetic fields and large scanners, fNIRS utilizes near-infrared (NIR) light to detect brain activation by measuring the associated hemodynamics. The portability of fNIRS positions it as a competitive imaging modality to address some of the challenges of conventional neuroimaging techniques, such as fMRI and magnetoencephalography, including a lack of wearability for continuous monitoring, limited temporal resolution, and need for subject immobility during use.^3^ It has shown great promise for safe and long-term monitoring of brain activity and is increasingly used in studies for behavioral^4^ and cognitive neurodevelopment,^5^^–^^8^ language,^9^^,^^10^ psychiatric conditions,^11^^,^^12^ stroke recovery,^13^ and brain–computer interfaces.^14^^–^^16^

Despite exponential growth in the number of applications^17^^,^^18^ and publications^3^ in recent years, many fNIRS systems still employ fiber-based cart-sized instrumentation^19^ that place limits on both channel density and the use of fNIRS in natural environments. Although fiber-based high-density^20^ and portable^21^ fNIRS systems have been demonstrated, the use of fragile fiber optics cables, stationary external source/detector units,^22^^,^^23^ and the need for individual and specialized headgear for specific tasks have motivated the fNIRS community to investigate more flexible modular and fiber-less designs.^24^^,^^25^

The modular fNIRS architecture is based on utilizing elementary optical source and detector circuits (modules) as repeating building blocks to form a re-configurable probe.^24^ This modular architecture offers significantly improved portability, scalability, flexibility in coverage, and fabrication cost.^24^ By avoiding the use of fragile optical fibers, modular fNIRS systems permit the use of light guides to directly couple light sources and detectors to the scalp, significantly reducing signal loss due to fiber coupling. The lightweight and compact modules also make wearable fNIRS and continuous monitoring in mobile environments possible.^3^^,^^26^ In addition, the ability to use both intra-module (within a single module) and inter-module (source and detector on different modules) channels allows for high-density probes with varying source-to-detector separations (SDS) that increase measurement density and tissue depth sampling, resulting in enhanced signal quality, and easy removal of physiological noise.^27^

Despite these perceived benefits, the task of designing a modular fNIRS probe can quickly grow in complexity as the number of modules increases. While parameters can be empirically determined when designing a single module, understanding the trade-offs among a large array of parameters, including module shape, module size, optode quantities, and optode locations, and each parameter’s effects on the final probe can become a daunting task. Not only do most published modular fNIRS studies largely focus on the design of a single module without addressing the effect of these module- and probe-level parameters on the final probe, the current literature also does not provide a means to compare probes composed of different module designs.

Aside from the challenges of determining these modular probe core parameters, other factors, such as mechanical, ergonomic, safety, usability, optoelectronic, and data communication considerations^24^ also play important roles in achieving the desired performance. For example, mechanical features such as optical coupling and electronic circuitry encapsulation must be considered alongside ergonomic considerations, such as comfort, weight, and robustness. Additionally, the use of high-density light sources in such modular probes brings about additional safety considerations, such as heat dissipation, driving voltage, and battery life. Moreover, optoelectronic considerations arise from the use of specialized optodes with narrow emission bandwidths, high gains, low noise, and fNIRS-optimized wavelengths. Not only are these specialized optodes more expensive due to their niche applications and characteristics, they also require more complex control electronics for driving optodes and acquiring data. With such dense coverage, complex encoding strategies such as frequency^28^ multiplexing become a necessity for obtaining high-density data acquisition to achieve sufficient spatial and temporal resolution. Finally, while previously reported modular fNIRS systems often employ daisy-chain communication protocols to connect multiple modules on a single bus,^29^^–^^33^ the design of physical inter-module connections,^34^ the synchronization method between modules,^24^ and the transfer of acquired data become increasingly complex with high module counts and branching connections.

Along these lines, a number of fNIRS data analysis packages exists.^35^^–^^37^ However, they focus on the statistical analysis of the data^35^^–^^37^ to enhance its quality and provide guidance on post-processing steps such as motion artifact correction.^35^ While some other tools exist to assist in the probe design,^38^^–^^41^ most of these tools are designed to work in a highly constrained design space, where the probe parameters are mostly pre-determined by the user. As a result, the best practices and trade-offs in modular probe design such as tessellation, connection, or re-orientation are poorly explored and understood. Therefore, the community is in great need of an easy-to-use software tool to assist the exploration of and quantitative comparisons among countless parameter choices in a modular probe design and to perform a limited degree of optimization within a well-constrained configuration.

A fully automated probe design and optimization pipeline is impractical without application-dependent design constraints. Instead, we report a simplified, easy-to-use software toolbox to help designers navigate the vast parameter space of a modular probe. We also share a number of fundamental modular probe design strategies, discovered through our explorations via this toolbox that were not widely recognized or previously studied. The entire workflow has been implemented into an open-source, MATLAB-based toolbox called Modular Optode Configuration Analyzer (MOCA).^42^ MOCA supports a list of commonly used module shapes, user-defined optode layouts, and region-of-interest (ROI) coverage, and can produce quantitative performance metrics, such as distributions of source–detector (SD) separations, sensitivity maps, and spatial multiplexing groupings. These performance metrics also allow comparisons between different designs of modular probes. Although MOCA is not designed as a fully automated software that produces “optimal” probes regardless of application, its unique capability to describe and sweep modular probe parameters in operator-guided interrogations offers valuable perspective to start approaching the complex modular hardware design problem and make informed comparisons between well-constrained design choices.

The remainder of the paper is outlined below. In Sec. 2, we discuss the relevant design considerations when developing a modular probe using MOCA. We specifically focus on the parameterization of the modules, processes required to assemble modules into functional probes, and related performance metrics for system characterization and comparisons. In Sec. 4, we demonstrate MOCA’s capability in designing full-head probes using a variety of module shapes and compare their trade-offs regarding channel density, SD separations, and average brain sensitivities. Furthermore, we utilize MOCA to showcase potential improvements to published fNIRS probes by altering module orientations, spacing, and staggering layouts. In Sec. 5, we highlight a number of generalizable design strategies that were discovered via our experiments using MOCA, including the importance of considering module orientations, tiling strategies, and module spacing tuning, among others.

## 2 Modular Probe Parameters and Performance Metrics

**Fig. 1:**
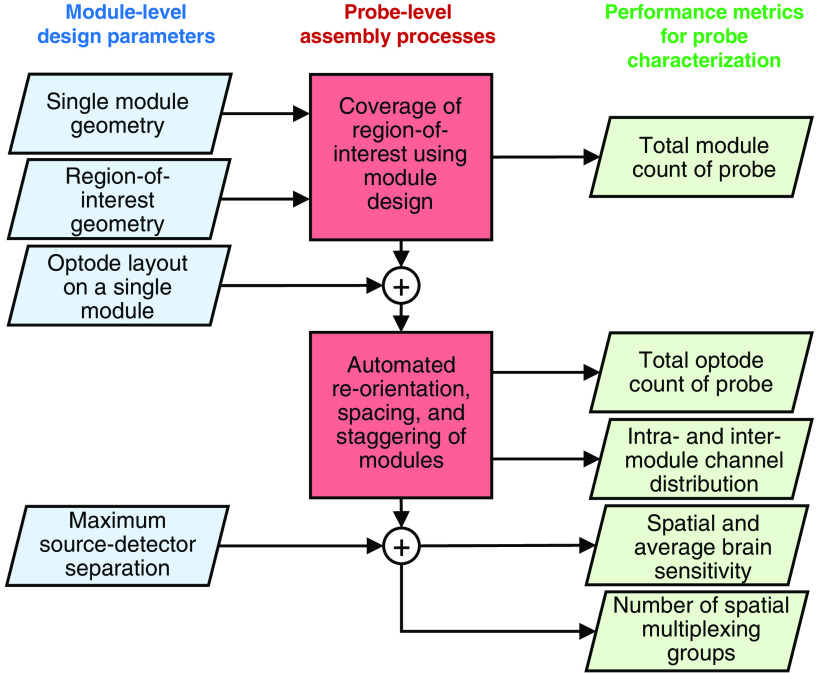
Workflow of module-level design parameters (left column; blue) used in probe-level processes (center column; red) to produce performance metrics to characterize a probe (right column; green). Performance metrics are organized top to bottom from least complex (two parameters needed) to most complex (four parameters needed). Arrows trace how parameters are used to derive specific performance metrics.

A diagram showing the overall design process of a modular fNIRS system is shown in Fig. 1. Specifically, the three parts describing MOCA’s workflow are (1) the design parameters describing a single module design; (2) the processes and parameters used to assemble the modules into a probe; and (3) the derived performance metrics used to characterize the resulting probe. MOCA starts with the definition of essential module parameters (shown in the left column in Fig. 1), applies those parameters along with probe-level constraints to a probe-generation process (center column in Fig. 1), and derives quantitative performance metrics of the resulting probe (shown in the right column in Fig. 1). Arrows in Fig. 1 define dependencies between the derived performance metrics and the input parameters. For example, to calculate the probe’s channel distribution, one must define the module geometry, ROI, and optode layout design parameters.

### 2.1 Essential Module-Level Design Parameters of fNIRS Modular Probes

The basic building block of a modular probe is an fNIRS module. It is typically in the form of an optoeletronic circuit made of a rigid^29^^,^^30^^,^^33^^,^^43^ or rigid-flex^44^^,^^45^ substrate with on-board light sources, optical sensors, auxiliary sensors, microcontrollers, and other communication electronics. A modular probe is subsequently constructed by replicating and interconnecting multiple identical modules. Therefore, the design decisions regarding the module-level parameters are highly important and directly impact the functionalities and restrictions of the resulting probe.

#### 2.1.1 Single module geometry

The shape of a module is one of the key parameters when designing a modular system. In published literature, simple polyhedral shapes, especially equilateral polygons (square, hexagon, etc), are typically used due to their simplicity to fabricate, analyze, and tessellate over a target ROI. It is also possible to design probes that combine multiple polygonal shapes, such as a combination of hexagonal and pentagonal modules. Such hybrid-shape modular systems may bring advantages in tessellating curved surfaces, but they also require more complex analyses. MOCA supports a number of built-in module shapes including three equilateral polygons (triangle, square, and hexagon). In such cases, the module edge length is the only shape parameter that needs to be defined. One should be aware that a small-sized module requires a large number of boards to cover a given area, thus resulting in higher fabrication cost and higher complexity in assembly and analysis. Moreover, a small module size also limits the maximum intra-module SDS. Shorter SD separations are known to be more sensitive to superficial tissues rather than brain activities. On the other hand, a small-module size provides better probe-to-scalp coupling when a rigid-board–based module is used. MOCA provides support for user-specified arbitrary polygonal modules, defined by a sequence of two-dimensional (2D) coordinates. Subsequent analyses of these user-defined arbitrary modules shapes only use the bounding box of these polygons when varying probe-level parameters.

#### 2.1.2 Target regions-of-interest

An ROI refers to the area of the scalp directly above the cortex for which brain activities are expected to occur.^46^ For simplicity, here, we focus on designing probes based on the coverage of a 2D ROI. For generality, MOCA specifies an ROI geometry as a closed polygon made of a sequence of 2D coordinates. Users need to specify at least three Cartesian coordinates to define a closed ROI. In the future, MOCA can potentially be expanded to support three-dimensional (3D) surfaces as ROIs through the use of 3D surface tessellation tools, such as the Iso2Mesh^47^ mesh generator and 3D photon transport modeling tools, such as NIRFAST^48^ and Monte Carlo eXtreme^49^ (MCX).

#### 2.1.3 Optode layout within a single module

Optode layout refers to the spatial arrangement of optical sources and light sensors within the boundaries of a single polygonal module. In MOCA, each source and detector position is defined by a set of discrete 2D coordinates relative to the module’s center. The 2D coordinates define the center of the active area of the light-emitting diode (LED), laser, or photo detector. The physical dimensions of the optodes as well as the size and location of electronic components needed to drive each optode are not considered. The SD separations between all combinations of SD pairs are derived based upon the optode positions.

#### 2.1.4 Maximum source–detector separation and maximum short separation channel

MOCA also considers the maximum SD separation (${\mathrm{SDS}}_{\max}$) as a key design parameter. Typically, ${\mathrm{SDS}}_{\max}$ is determined by the signal-to-noise ratio of the detected signal.^50^ A large SDS has low detector sensitivity due to the exponential decay of light intensity as SDS increases. This maximum separation limits the number inter-module channels that emerge from a particular tessellation of modules over an ROI. By default, MOCA considers any SDS below 10 mm to be a short-separation (SS) channel. This threshold can be manually changed to fit any specific optode performance or probe application. MOCA uses 30 mm as the default ${\mathrm{SDS}}_{\max}$.^51^^,^^52^ MOCA bounds the SD range by the SS channel threshold and the ${\mathrm{SDS}}_{\max}$.

### 2.2 Probe-Level Assembly Process Parameters

A modular probe is constructed when multiple modules are arranged to form a non-overlapping coverage of the ROI area. The final probe is dependent on the tessellation (the number of modules and the spacing between them) and the orientation of each individual module in the probe.

#### 2.2.1 Exploring module tessellation and probe spacing

MOCA provides a process to tessellate modules over a user-defined 2D polygonal ROI, which is generally known as the “tiling” problem in computational geometry.^53^ Here, a “complete tessellation” refers to the tiling of an ROI using a single module shape without overlapping or leaving a gap in coverage. Each of the three built-in polygons (triangle, square, and hexagon) has the ability to cover a 2D area.^54^ MOCA performs the tessellation by first tiling the module shape along a horizontal axis starting at the lowest vertical coordinate of the ROI until the width of the row composed of adjacent modules is wider than the width of the corresponding segment of ROI the row is tiled over. It then repeats this row-generation process until the height of all the rows combined is larger than the maximum height of the defined ROI. This dimension comparison in both axes accounts for module shapes with non-vertical and non-horizontal sides. For irregular module shapes, MOCA uses the maximum width and maximum height of the defined polygon as the bounding box to create a tiling grid of the module over the ROI. Using the maximum width and height of the ROI as a guide for tiling ensures the full ROI is covered. Although MOCA offsets and flips the three equilateral polygon shapes to prevent gaps, irregular module shapes have inherent gaps between modules when tessellated. Additionally, MOCA accepts manually defined tessellations by reading a sequence of coordinates defining the center of modules to specify each individual module’s location within the ROI. Following tessellation, each module is assigned a unique index and an adjacency matrix is constructed to represent which modules are next to one another.

To extend the flexibility of probe creation, users can change probe spacing, the minimum distance between adjacent modules in all directions. Additionally, a module can be manually deleted from the tessellation to allow the probe to more closely follow the boundaries of the ROI or better represent intentional empty spaces in the probe. When individual modules are removed from the probe, the adjacency matrix is re-calculated from the resulting probe.

#### 2.2.2 Guiding module orientation and connection routing

Module orientation refers to the rotation of the module along the normal direction of the ROI plane. In a complete tessellation of the three equilateral polygon shapes, MOCA appropriately flips and translates modules to prevent gaps and overlaps. For tessellations of irregular shapes, each module is simply placed in the same orientation as it was originally defined. After probe generation, MOCA allows the user to manually change the orientation of individual modules based on their assigned indices. For asymmetric optode layouts, changing the module orientation alters the SDS of inter-module channels, resulting in different performance metrics.

Additionally, MOCA creates a single sequential path to connect all modules to form a linear data communication bus, referred to as the “routing” process. In such a path, all modules are connected and every module is visited exactly once—a classic problem known as the Hamilton path^55^ in graph theory. In most configurations, a Hamilton path is not unique, and computing such a path is known to be an NP-hard problem, i.e., problems that do not have a polynomial complexity when the node number grows. However, due to the limited module numbers commonly used in an fNIRS probes, an exhaustive search of the adjacency matrix can typically identify all Hamilton paths in a given tessellation with no more than a few minutes of computation. For any computed path, MOCA then orients each module based on the angle of a vector defined by the center of the oriented module and the center of the following module in the path. The orientation angle is relative to the horizontal axis.

### 2.3 Performance Metrics to Characterize Probes

Each metric described below changes as module- and probe-level parameters are altered either manually or through MOCA’s sweeping functions. MOCA not only helps unravel the complex interplay between choices of different parameters, but also guides the probe designer in making trade-offs between conflicting design targets—improving one metric may come at the risk of worsening another. We have chosen the following set of essential performance metrics due to their ability to easily inform a breadth of end-user probe requirements such as cost, weight, depth sensitivity, and sampling rate estimates.

#### 2.3.1 Total module and optode counts

Based on the module design and tessellation, MOCA computes the total number of modules, $n_{m}$, needed to cover the ROI. In addition, MOCA also outputs the total number of sources ($n_{s}$) and detectors ($n_{d}$) of the final probe. All modules, sources, and detectors of an assembled probe are given unique identifiable index numbers ($m_{i}$, $s_{i}$, and $d_{i}$, respectively). Module and optode counts are performance metrics outputted by MOCA from which cost, weight, and power estimates can be deduced.

#### 2.3.2 Inter- and intra-module channel distribution

For any assembled probe, MOCA generates histograms of the SD separations for all combinations of SD pairs. Particularly, it outputs separately the distribution of inter- and intra-module channels that are below the ${\mathrm{SDS}}_{\max}$ previously defined by the user. These channel distributions aid the user in designing the probe based on the targeted application and population. For example, shorter channels are more applicable to infant populations. Additionally, MOCA outputs channel density, a metric commonly used for fNIRS probe bench marking. Channel density is defined as the number of channels, $n_{\text{channels}}$, divided by the area of the ROI.^24^ Furthermore, MOCA can provide a spatial plot overlaying channels on the assembled probe, allowing for visual inspection of low channel density areas within the probe.

#### 2.3.3 Spatial brain sensitivity

Brain sensitivity ($S_{\text{brain}}$) refers to the magnitude of the measurement signal change at a detector given a localized perturbation of optical properties of brain tissue.^56^ A higher $S_{\text{brain}}$ value suggests the probe is more sensitive to the anticipated brain activation. It is calculated from the spatial probability distribution of photons scattering through complex tissue as they travel from the source to the detector.^57^ Although modeling 3D head/brain anatomies and 3D-based light simulations have been reported, including several related works from our group,^47^^,^^49^^,^^58^^,^^59^ we deliberately chose a simplified layered-slab head model and 2D based probe layout as default models to evaluate a modular probe in MOCA. Such a decision was largely motivated by (1) significantly faster computation and pre-/post-processing to accommodate fast sweeping of a large parameter space, and (2) avoiding another added layer of complexity when probe design is coupled with underlying brain anatomy in a 3D head model. A comparison between $S_{\text{brain}}$ computed by 2D and atlas-based analyses is provided in Sec. 4. Nonetheless, MOCA can export 2D probe data to established 3D probe modeling toolkits, such as AtlasViewer^41^ and MCX,^49^ to perform more advanced analyses when 3D head models are necessary.

MOCA uses a five-layer slab model consisting of tissue imitating the scalp, skull, cerebral spinal fluid (CSF), white matter (WM), and gray matter (GM) to determine the spatial sensitivity profile for each SD pair in a probe.^60^ The thickness of each tissue layer in the slab is set to the average thickness of that tissue type computed using the top half of a tetrahedral brain model.^61^ We define the brain region as the combination of GM and WM tissues. The optical properties and resulting thicknesses for each tissue type are summarized in Table 1.

**Table 1:** Optical properties used in the slab model for calculating brain sensitivity based on Fang et al.^58^ The thickness of each layer is derived by dividing the total tissue volume by the tissue’s surface area from a tetrahedral five tissue brain model.^61^ The absorption coefficient, $\mu_{a}$, is the average path a photon will travel in the medium before being absorbed. Similarly, the scattering coefficient, $\mu_{s}$, defines the average path length of photons before a scattering event. Anisotropy, g, is a unit less measure of the amount of forward direction retained after a single scattering event.

Tissue type	$\mu_{a}$ (${\mathrm{mm}}^{-1}$)	$\mu_{s}$ (${\mathrm{mm}}^{-1}$)	g	Thickness (${\mathrm{mm}}^{-1}$)
GM	0.020	9.000	0.89	7.25
WM	0.080	40.900	0.84	4.00
CSF	0.004	0.009	0.89	2.73
Skull	0.019	7.800	0.89	3.29
Scalp	0.019	7.800	0.89	4.23

For each SD pair in the assembled probe, $3\times 10^{8}$ photons are simulated using our in-house 3D Monte Carlo photon transport simulator, MCX,^49^ using a pencil beam source and a single 1.5-mm radius detector placed at the surface of the slab at its corresponding SDS. In a voxelated grid, $S_{\text{brain}}$ is defined as a ratio dividing the region-wise summation of the sensitivity matrix in each brain tissue region by the summation of the entire sensitivity matrix for each source–detector separation,^57^ i.e.,

$$S_{\text{brain}}(s,d)=\frac{\underset{r\in \Omega_{GM}}{\sum }J(r,s,d)+\underset{r\in \Omega_{\mathrm{WM}}}{\sum }J(r,s,d)}{\underset{r\in \Omega }{\sum }J(r,s,d)},$$ (1)

where the sensitivity matrix, also known as the Jacobian (J), is computed using the adjoint Monte Carlo method.^62^ In addition to $S_{\text{brain}}$, MOCA also calculates the average brain sensitivity for the entire probe, $\overset{¯}{S_{\text{brain}}}$, based on all the SD separations above the SS threshold. SS channels are excluded in the calculation of $\overset{¯}{S_{\text{brain}}}$ because, by definition, they are designed to only sample superficial layers.^57^

#### 2.3.4 Spatial multiplexing groups

The density of assembled modular probes may impact the probe’s temporal sampling rate when illuminating each source sequentially. MOCA introduces spatial multiplexing, an encoding strategy that can potential accelerate data acquisition by simultaneously turning on multiple light sources at the same time. Because of the high attenuation of light in the head and brain tissues at large separations, MOCA can ignore the cross-talk of light sources that are far away from a given detector and assign sources into spatial multiplexing groups, or SMG, so that all sources within an SMG can be turned on simultaneously. By default, MOCA uses the ${\mathrm{SDS}}_{\max}$ as the minimal distance between sources. This distance, however, can be defined by the user. Notably, unlike frequency multiplexing, spatial multiplexing does not require extra energy-intensive hardware or post-measurement separation of combined signals.

The search for the SMG starts by randomly specifying a source position as the seed; a circle of radius ${\mathrm{SDS}}_{\max}$ centered at the seed position is drawn and a random source outside of this circle that is at least $2\times {\mathrm{SDS}}_{\max}$ away is picked; the above process repeats until no additional source can be found. Once an SMG is identified, a new source that does not belong to any existing SMG is selected as the new seed for the next SMG and the above process repeats until every source is allocated. The total number of spatial multiplexing groups, $n_{\mathrm{SMG}}$, depends on the tessellation of the module over the ROI as well as the choice of the seed position. As with channels, the $n_{\mathrm{SMG}}$ are for a single wavelength. Thus, when estimating the total sampling rate of the probe using dual-wavelength sources, the control unit must cycle through each group twice (once for each wavelength).

In addition to $n_{\mathrm{SMG}}$, MOCA calculates the spatial multiplexing ratio (SMR), defined as $\mathrm{SMR}=n_{s}/n_{\mathrm{SMG}}$. This ratio is interpreted as the acceleration factor of the data acquisition speed when using spatial multiplexing. For example, for a 20-source probe, an $n_{\mathrm{SMG}}$ of 5 can accelerate the data acquisition by a factor of SMR=20/5=4 fold.

## 3 Additional Functionalities

MOCA was created as an exploratory tool to interrogate specific design parameters and reveal the trade-offs, within a well-constrained search space, regarding specific design decisions. MOCA possess functions to facilitate changing probe-level parameters and exporting the desired probe for use in existing probe design tools such as AtlasViewer.

### 3.1 Parameter Sweeping

#### 3.1.1 Altering spacing between modules

An optional parameter during module tessellation is probe spacing—a uniform distance assumed between adjacent modules. The spacing sweep function varies the probe spacing within a user-defined range in user-defined increments. For the three built-in polygons (triangle, square, and hexagon), spacing is increased between all adjacent sides of the modules within the probe. For arbitrary shapes, spacing is added to the horizontal and vertical sides of the rectangular bounding box. The number of modules required to cover the ROI is continuously adjusted as probe spacing is varied. The performance metrics for each of the resulting probes are reported by MOCA as a function of probe spacing.

#### 3.1.2 Exhaustive search of module orientations

MOCA provides a limited orientation enumeration function to re-orient modules through a predefined number of orientations. For the three built-in polygons, the default number of re-orientations per module is simply the number of sides of the polygon. For arbitrary shapes, the default number of re-orientations is four based on the bounding box. Additionally, a user can describe the number of orientations for any shape. MOCA re-orients modules in evenly spaced angle increments. An exhaustive search is performed using the number of modules in the probe and the number of user-defined orientations. Each probe resulting from each permutation of module re-orientations is characterized by MOCA and reported as a function of various probe layouts.

#### 3.1.3 Staggering rows of modules

Staggering modules refer to shifting a row (or column) of tessellated modules in the x (or y) axis. Staggering is performed on tiling grid probe layouts. Adjusting this probe-level parameter is particularly useful for improving probes composed of modules with symmetrical optode layouts, where re-orienting modules does not affect SDS, or when high-density probes are needed, where probe spacing cannot be increased. A user defines both the range and increment by which to offset a particular row. Each resulting probe is analyzed and the corresponding performance metrics are calculated. MOCA then reports a plot of the $\overset{¯}{S_{\text{brain}}}$, SMR, and the number of channels for each staggered probe.

### 3.2 Exporting Probe for Use in AtlasViewer

**Fig. 2:**
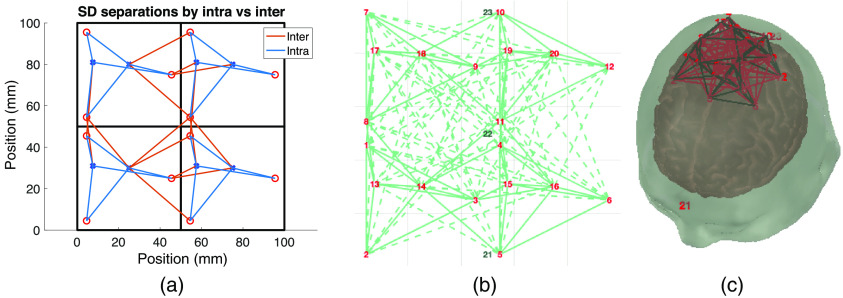
Example probe exported for use in AtlasViewer.^41^ (a) A four-module probe with three sources (red circles) and two detectors (blue crosses) plotted using MOCA. Intra- (blue) and inter-module (orange) channels are shown in solid lines. (b) Imported probe in SDgui. Solid lines represent fixed springs. Dashed green lines represent flexible springs between sources and detectors. Three dummy optodes (numbered 21, 22, and 23) are shown in black. (c) The resulting probe in AtlasViewer registered to an atlas using the dummy optodes as anchors.

MOCA performs its analysis of module- and probe-level parameters on an infinite slab model derived from the Colin27 atlas. When 3D analysis is desired, MOCA can export the probe layout to a “.sd” file for use in “SDgui” — a built-in tool of AtlasViewer^41^ used for creating and editing .sd files. To properly represent a modular probe layout in AtlasViewer (which treats all optodes individually without a reference to a module), MOCA first translates the module-level parameters by creating fixed/rigid springs between all optode pairs (source–source, source–detector, and detector–detector) within each module. These fixed springs maintain the relative optode layout within each module while permitting bending at the junctions between springs. MOCA then adds fixed springs between each inter-module channel (SD pairs between modules with distances below the ${\mathrm{SDS}}_{\max}$) to translate the probe-level parameters (spacing, orientation, staggering). As an additional constraint, MOCA adds flexible springs (springs of length -1) for inter-module channels above the ${\mathrm{SDS}}_{\max}$. Finally, to register the probe to the surface of the selected atlas, MOCA adds three dummy optodes to the exported “.sd” file. All three optodes are placed at the midpoint between the minimum and maximum x coordinates of all optodes in the probe. The y coordinate of the first, second, and third dummy optodes are set to the minimum y coordinate, midpoint, and maximum y coordinate of all optodes in the probe, respectively. The first, second, and third dummy optodes are assigned to the Fpz, Cz, and Oz positions, respectively, in the standard 10–10 system. This places any MOCA-designed probe at the top of an atlas by default. A user can change the dummy optode anchors to re-position the probe on an atlas. The exported .sd file can then be loaded into AtlasViewer for placement on a generic or subject-specific atlas (Fig. 2).

## 4 Results and Practical Examples

In this section, we first validate the $S_{\text{brain}}$ derived from a simplified five-layer slab model against previously published atlas-based $S_{\text{brain}}$ results.^56^ Then we demonstrate how the module-level parameters of MOCA can be used to characterize and compare full-head probes composed of different choices of elementary module designs. Lastly, we show examples using MOCA’s assembly processes as investigational tools to potentially improve existing designs by altering probe-level parameters, such as probe spacing, module orientations, and the staggering of modules within an assembled probe.

### 4.1 Slab-Based Brain Sensitivity Corresponds with Atlas-Based Sensitivity

**Fig. 3:**
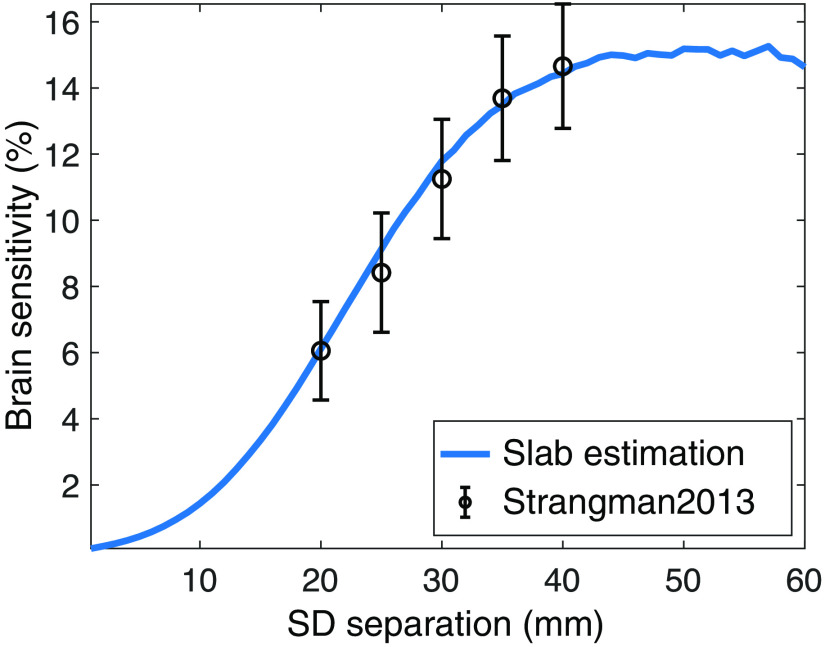
Results comparing brain sensitivity derived from finite slab models used by MOCA and atlas-based models. The blue line shows calculated brain sensitivity based on a five-layer slab model for SD separations from 0 to 60 mm in 1-mm increments. Overlaid in black are the brain sensitivity results calculated from an atlas by averaging brain sensitivity for fixed source–detector separations across nineteen locations in the international 10–20 system.^56^

Figure 3 shows $S_{\text{brain}}$ calculated using our five-layer slab model at SD separations ranging from 1 to 60 mm in 1-mm increments (blue line). We also overlay full-head averages of $S_{\text{brain}}$ and standard deviations at 20, 25, 30, 35, and 40 mm separations from a previously published study^56^ using the Colin27 atlas.

Simulations on a five-layer slab model show an increase in $S_{\text{brain}}$ as SDS increases. Additionally, $S_{\text{brain}}$ for SD separations below 10 mm is <1.17%. At 20-, 25-, 30-, 35-, and 40-mm separations, the maximum difference between the atlas-based and slab-based $S_{\text{brain}}$ values is <0.6%. Figure 3 shows that using 2D approximation of the ROI and a layered brain structure provides a reasonable trade-off between accuracy and computational efficiency, especially for high-density probe characterization.

### 4.2 Comparison between Sample Modules of Various Shapes

MOCA allows the comparison of a wide range of fNIRS module designs by quantifying the effects of probe-level design parameters on a probe’s performance. As a showcase, here we report the results from a comparison of three equilateral module shapes (square, hexagon, and triangle) with the same optode layout tessellated over a 200×200  mm ROI, derived from the average surface area of the top half of an adult male head.^63^ Square^29^^–^^31^ and hexagonal^32^^,^^33^^,^^43^ fNIRS modules have been extensively studied in literature and are chosen here for a quantitative comparison. While an equilateral triangle has not been reported in published module designs, we include it here because of the potential suitability for better tessellation of a 3D surface in future extensions. With this comparison, we want to demonstrate both the scalability of MOCA in analyzing full-head probes and how performance metrics change across module-level design decisions.

**Fig. 4:**
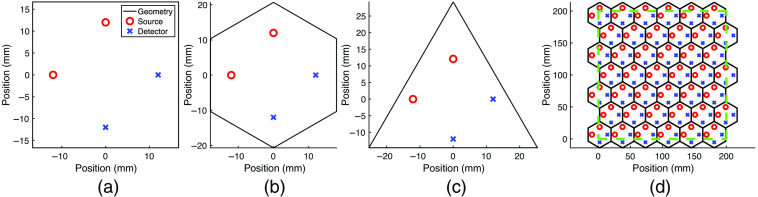
Elementary module designs used in a full-head comparison. (a)–(c) The perimeter of the square, hexagon, and triangle-based module designs, respectively. The optode layout of all three shapes is identical. Red circles represent sources while blue crosses represent detectors. (d) Tessellation of the hexagon module over an ROI. The dashed green line outlines the 200×200  mm ROI.

As mentioned above, MOCA systematically tessellates the target ROI using the module geometry and assigns each module an index number. If not considering within-module optode locations, only translation is needed for both square and hexagon modules to completely cover a region. For the triangle shape, MOCA rotates every other triangle and its optodes 180 deg to fill the ROI without leaving any gaps. No other probe-level parameter changes are made for this comparison. Probe spacing is set to zero. The default SS threshold is set to 10 mm, and the ${\mathrm{SDS}}_{\max}$ is set to 30 mm. The minimum distance between sources used in calculating SMGs is set to $2\times {\mathrm{SDS}}_{\max}$. To avoid simultaneously changing multiple parameters and only focus on module shape, an identical optode layout made of two sources and two detectors is used in all three module designs in this example. The edge-length of the square is set to 33.33 mm, determined by the average length of three previously reported square-shaped module designs.^29^^–^^31^ The edge-length of the hexagon and triangle is set to 20.68 and 50.65 mm, respectively, calculated to achieve the same area as the square module. The three module designs as well as the tessellation of the hexagon-based probe over the ROI are shown in Fig. 4. The derived performance metrics for each of the three sample probes are summarized in Table 2. The results that follow are only applicable for the specific module- and probe-level parameters chosen for this showcase.

**Table 2:** Summary of quantitative performance metrics derived by MOCA when tessellating the three elementary module shapes over a 200×200  mm region of interest.

Row	Performance metric	Square-based probe	Hexagon-based probe	Triangle-based probe
1	Total modules (N)	36	42	40
2	Total optodes (N)	144	168	160
3	Total channels (N)	324	405	496
4	Intra-module channels (N)	180	237	336
5	Inter-module channels (N)	144	168	160
6	% of channels that are inter-module (%)	55.56	58.52	67.74
7	Average brain sensitivity (%)	7.52±1.95	6.50±2.44	8.83±3.10
8	Average intra-module brain sensitivity (%)	6.44±2.10	6.44±2.10	6.44±2.10
9	Average inter-module brain sensitivity (%)	8.82±0.00	6.54±2.66	9.94±2.86
10	Spatial multiplexing groups (N)]	9	8	13
11	Spatial multiplexing Ratio	8	10.5	6.15

#### 4.2.1 Effect of module shape on channel separation distributions

**Fig. 5:**
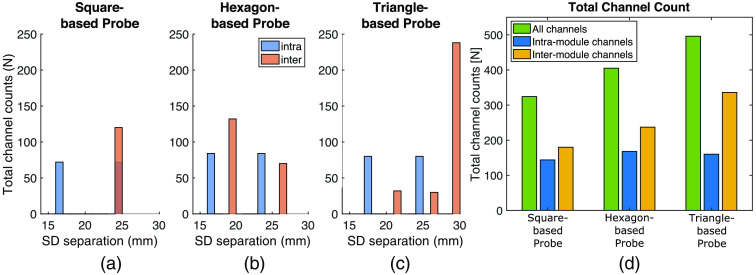
Channel distributions and total channel counts resulting from the tessellation of the three elementary module shapes over a 200×200  mm region of interest. (a)–(c) Resulting intra- and inter-module channel distributions for square, hexagon, and triangle module-based probes. (d) The total channel count of each probe grouped by intra- and inter-module channels.

Figure 5 shows a histogram of the SD separations of the full-head (200×200  mm area) probe composed from the three selected module shapes. Table 2 shows that the number of modules needed to cover the ROI varies for each shape due to the complete coverage constraint enforced by MOCA for this showcase [Fig. 4(d)]. Since each module utilizes the same optode layout, the intra-module channel distributions [blue bars in Figs. 5(a)–5(c)] are simply scaled by the total numbers of modules needed to completely cover the ROI. The SDS of inter-module channels are dependent on the module shape, resulting in varying inter-module channel distributions between all three probes [orange bars in Figs. 5(a)–5(c)].

For this particular example, the triangle-based probe reports both the highest number of total channels [Fig. 5(d)] and the largest SD separations of all three tessellated probes [Fig. 5(c)]. The hexagon-based probe appears to have the shortest inter-module channels [Fig. 5(b)]. Due to its symmetry and given the ${\mathrm{SDS}}_{\max}$ setting, the square-based probe happens to have all SD separations at 24 mm. Notably, the triangle-based probe adds the most inter-module channels, almost twice the number of intra-module channels [Fig. 5(d)], while also requiring two fewer modules than the hexagon-based probe (Table 2, Rows 1–5). Figure 5(d) also shows that the number of inter-module channels is greater than the number of intra-module channels for all three probes.

#### 4.2.2 Combining intra- and inter-module channels for brain sensitivity

**Fig. 6:**
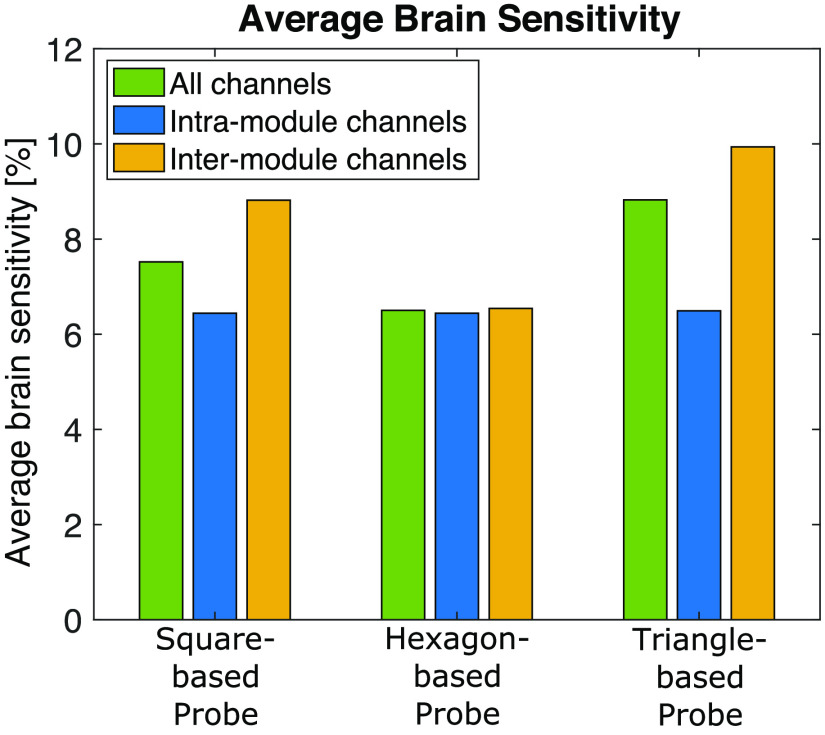
Resulting average brain sensitivity organized by intra- and inter-module channels for square-, hexagon-, and triangle-based probes tessellated over a 200×200  mm region. SS channels are excluded in all calculations.

The $\overset{¯}{S_{\text{brain}}}$ values derived from the three probe designs, grouped by intra-module channels, inter-module channels, and all channels, are summarized in Fig. 6. Only channels above the SS threshold and below the ${\mathrm{SDS}}_{\max}$ are used. Despite having the fewest total channels (Table 2, Row 3), the square-based probe results in a higher $\overset{¯}{S_{\text{brain}}}$ than the hexagon-based probe. For the square- and triangle-based probes, the use of inter-module channels increases the probe’s $\overset{¯}{S_{\text{brain}}}$ as compared to simply using intra-module channels alone. For the hexagon-based probe, $\overset{¯}{S_{\text{brain}}}$ computed using only intra-module channels is similar to that when using only inter-module channels (6.44% versus 6.54%). Due to having the same optode layout, the intra-module $\overset{¯}{S_{\text{brain}}}$ is the same for all three probes.

#### 4.2.3 Effect of module shapes on improving sampling rate

**Fig. 7:**
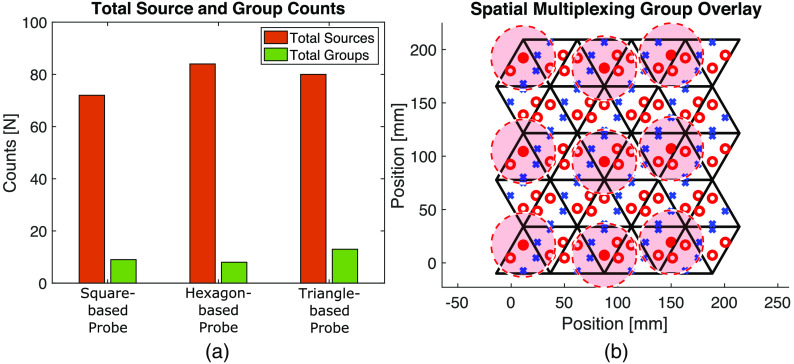
Spatial multiplexing group results from the tessellation of the square-, hexagon-, and triangle-based probes. (a) Comparison of total number of sources (orange) and total number of spatial multiplexing groups (green). (b) The triangle-based module tessellation with sources (red circles) and detectors (blue crosses). The dashed red circles indicate the effective region (30-mm radius) of each of the nine sources in the first spatial multiplexing group. The nine sources turned on simultaneously in this group are indicated by filled in red circles.

The total $n_{s}$ compared to the $n_{\mathrm{SMG}}$ arising from the tessellation of each module over the ROI are compared in Fig. 7(a). The total number of sources for the square-, hexagon- and triangle-based probes are 72, 84, and 80, respectively. Figure 7(b) overlays the first SMG over the triangle-based full-head probe. Using the $n_{\mathrm{SMG}}$ for each probe (Table 2, Row 10), the SMR (the ratio between $n_{s}$ and $n_{\mathrm{SMG}}$) is 8, 10.5, and 6.15 for the square-, hexagon-, and triangle-based probe, respectively. This result indicates that the hexagon-based probe’s sampling rate can benefit the most when using group-based spatial multiplexing.

### 4.3 Improving Existing Probes through Probe-Level Parameter Alterations

The ability to compute performance metrics from basic design parameters allows users to explore probe-level alterations and potentially improve existing probes using MOCA. Here, we simulate and alter published examples to demonstrate how even simple module layout changes such as rotating selected modules, altering probe spacing, and staggering modules can potentially improve published probe designs.

#### 4.3.1 Effect of optode orientation on probe characteristics

**Fig. 8:**
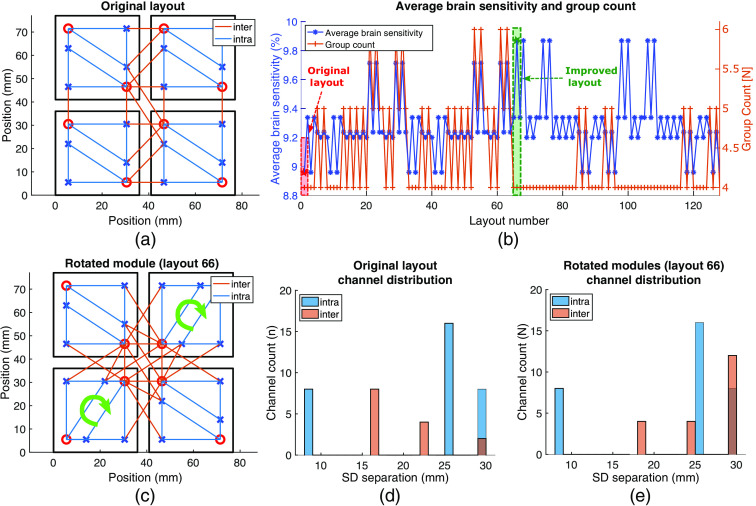
A four-module probe simulated using MOCA. (a) All modules are oriented in the same direction. Red circles represent sources and blue crosses represent detectors. An exhaustive search of all combinations of orientations for each of the four modules results in 256 possible layouts. The average brain sensitivity and number of spatial multiplexing groups for the first 128 layouts are shown in (b). The original layout (layout number 1) is highlighted in the red square. An example layout with the maximum possible brain sensitivity (layout number 66) is highlighted in the green square. (c) A visual representation of layout 66 with the bottom-left and top-right modules rotated 90 deg clockwise with respect to orientation in (a). Intra- and inter-module channel distribution resulting from the original layout is shown in (d). Channel counts resulting from the probe configuration in (c) are shown in (e). (d) and (e) In both channel distribution histograms, intra- and inter-module channels are shown in blue and orange, respectively. Dark orange indicates overlapping histogram counts.

Re-orienting modules within existing probes alters the SDS distribution and, consequently, the probe’s $S_{\text{brain}}$ and SMR. In Fig. 8, we simulate a $36\;{\mathrm{mm}}^{2}$ square module in a probe configuration inspired by the μNTS fNIRS module described in Chitnis et al.^29^ The modules in the initial tessellation are oriented in the same direction as in the original paper [Fig. 8(a)]. In our investigation, the spacing between each module is set to 5 mm and the ${\mathrm{SDS}}_{\max}$ is set to 30 mm. Each module has two sources and four detectors, resulting in 8 intra-module channels per module ranging from 8 to 29 mm. A total of 256 different probe configurations result from exhaustively re-orienting each module individually by 90 deg. Without losing generality, a subset of 128 layouts are shown in Fig. 8(b) to show the range of the variations.

Of the 256 possible layout configurations, eight of those layouts result in a maximum average brain sensitivity of 9.87%. These eight layouts also achieve the minimum number ($n_{\mathrm{SMG}}=4$) of spatial multiplexing groups. The intra- and inter-module channel distribution and channel count resulting from the MOCA analysis of the original probe layout are shown in Fig. 8(d). Figure 8(c) shows the same four-module probe but constructed with the bottom-left and top-right modules rotated 90 deg clockwise, corresponding to layout number 66 in Fig. 8(b). Using MOCA, the spatial channel plot overlaid onto this re-oriented probe shows a denser coverage of the center of the ROI compared to the original probe layout. The channel count distribution of this re-oriented probe is shown in Fig. 8(e). As expected, the intra-module channels in Figs. 8(a) and 8(c) are identical. However, re-orienting the two modules produces a shift toward longer separation inter-module channels that are known to be more sensitive to brain tissues. The number of inter-module channels within the 10- to 20-mm range decreases from 8 to 4 and the number of 29-mm separation inter-module channels increases from 2 to 12 upon re-orienting the two modules. The re-orientation of modules not only allows the probe to have more long-separation (LS) channels, it also increases the total number of inter-module channels from 14 to 20 [Figs. 8(d) and 8(e)]. Additionally, $\overset{¯}{S_{\text{brain}}}$ of the probe increases from 8.56% to 9.87% [Fig. 8(f)] while the number of spatial multiplexing groups, and subsequently the probe’s sampling rate, remains the same.

#### 4.3.2 Effect of probe spacing on probe performance

**Fig. 9:**
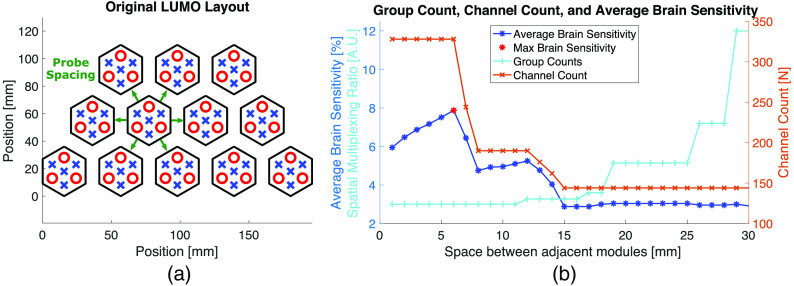
An analysis of hexagonal modules in a twelve-module probe. (a) Green arrows indicate the distances between modules as probe spacing varies. (b) The total channel count, average brain sensitivity, and the SMR at probe spacing values between 1 and 30 mm. Module orientations are held constant.

Probe spacing—the distance between edges of adjacent modules in a probe—is a parameter that can vary the resulting channel distribution and channel density of a probe by altering the relative distances between optodes on neighboring modules. To investigate the effect of this parameter, in Fig. 9, we simulate the probe layout described by Zhao et al.,^33^ which utilizes hexagonal shaped LUMO fNIRS modules developed by Gowerlabs.^64^ The length of each side of the hexagonal-shaped module used in our investigation is set to 18 mm and each module contains three sources and four detectors. The ${\mathrm{SDS}}_{\max}$ is set to 30 mm. A uniform spacing is set between all adjacent modules. Probe spacing is varied from 0 to 30 mm in 1 mm increments.

When all modules are densely packed with a spacing of 1 mm, the probe results in 328 total channels (184 of which are inter-module channels), an $\overset{¯}{S_{\text{brain}}}$ of 5.95%, and 12 SMGs. When the probe spacing is increased to 6 mm, the number of channels and spatial multiplexing groups remain the same while the $\overset{¯}{S_{\text{brain}}}$ increases [Fig. 9(b)]. The increase in $\overset{¯}{S_{\text{brain}}}$ arises due to the overall increased distances between sources and detectors of inter-module channels which sample deeper into the brain tissue. This results in a local maximum $\overset{¯}{S_{\text{brain}}}$ of 7.87%.

When we increase probe spacing to 8 mm, the inter-module channel separations increase to above the ${\mathrm{SDS}}_{\max}$. This decreases the number of usable inter-module channels and the probe’s $\overset{¯}{S_{\text{brain}}}$. The SMR remains unchanged between 6- and 8-mm probe spacing. Above 11 mm, the increase in probe spacing increases the relative distance between adjacent sources, allowing more sources to be turned on at the same time and decreasing the $n_{\mathrm{SMG}}$ needed. This trend continues as we increase probe spacing. Consequently, the probe’s $\overset{¯}{S_{\text{brain}}}$ reaches a minimal plateau of 3% at 15 mm spacing and beyond because only intra-module channels above the SS threshold remain within the SD range [Fig. 9(b)]. Similarly, since modules are further apart, the $n_{\mathrm{SMG}}$ continues to drop which increases the SMR (and the sampling rate of the probe when spatial multiplexing encoding is utilized). At 29-mm spacing, the SMR value is 12 due to only three spatial multiplexing groups needed (one for each of the three sources on each module).

#### 4.3.3 Effect of staggering modules on probe characteristics

**Fig. 10:**
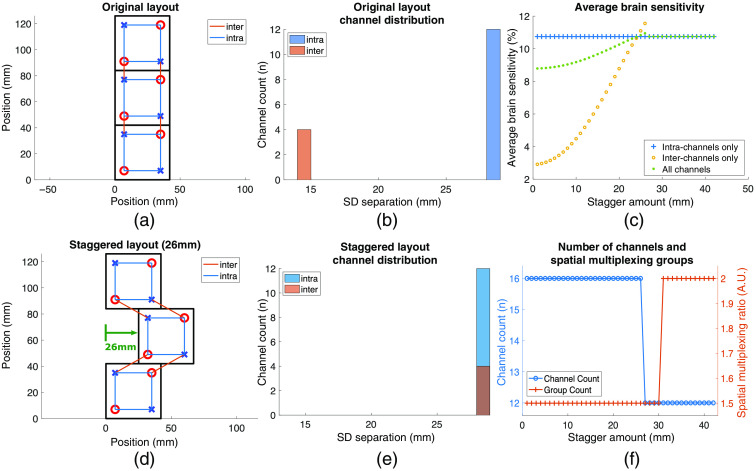
An analysis of square modules in a three-module probe. (a) A traditional three-module tessellation. Red circles represent sources and blue crosses represent detectors. (b) The resulting intra- and inter-module channel distribution from the probe layout in (a). (c) The average brain sensitivity for each layout resulting from module staggering separated by intra- and inter-module channel contributions. (d) The center module staggered by 26 mm, resulting in increased channel separation for inter-module channels, as shown in (e). (f) The total channel count and the number of spatial multiplexing groups of the probe layout as the center module is staggered between 0 and 42 mm.

Staggering adjacent modules within a high-density probe can increase inter-module SD separations to improve performance. To demonstrate the effect of staggering on the resulting probe, in Fig. 10 we simulate a $42\;{\mathrm{mm}}^{2}$ square module in a 3×1 layout configuration inspired by M3BA modules.^30^ Each of our simulated modules contain two sources and two detectors. The probe was staggered by translating the center module between 0 mm and 42 mm along the horizontal axis.

In Fig. 10(a), we overlaid the intra- (blue) and inter-module (orange) channels over the three-module probe. The resulting channel distribution shows 12 intra-module channels at 28 mm and 4 inter-module channels at 14-mm SD separations [Fig. 10(b)]. The $\overset{¯}{S_{\text{brain}}}$ of this probe using all channels is 8.79% [Fig. 10(c)]. When analyzed separately by intra- and inter-module channels, the $\overset{¯}{S_{\text{brain}}}$ using only intra-module channels (10.75%) is larger the $\overset{¯}{S_{\text{brain}}}$ when using only inter-module channels (2.9%) since in this tessellation intra-module channels are larger and probe deeper into the tissue.

In Fig. 10(c), we show the effect of staggering the tessellated module layout by translating the center module along the horizontal axis. This alteration increases the inter-module channel separations. Consequently, the $\overset{¯}{S_{\text{brain}}}$ due to only inter-module channels increases until the inter-module channel separations are larger than the ${\mathrm{SDS}}_{\max}$. The $\overset{¯}{S_{\text{brain}}}$ using all channels increases from 8.79% in the original tessellation to a maximum of 10.95% in the staggered tessellation at 26 mm. The $n_{\mathrm{SMG}}$ between the two layouts remained the same until a staggering amount of 31 mm at which point the sources are far away enough to group them together [Fig. 10(f)].

## 5 Discussion

Through the case studies shown in Sec. 4, we demonstrate the high complexity in designing a modular probe, where even adjusting a single parameter may have a profound impact on other parameters and the overall performance. Despite the fact that MOCA only permits operator-guided parameter interrogation in a well-constrained problem, the results from the above experiments did reveal a number of important design strategies that were not previously discussed in literature, including the effect of module re-orientation, fine-tuning the space between modules, and module staggering to potentially improve existing fNIRS probes.

Figure 5 reveals that despite having the same optode layout, probes composed of different module shapes covering the same ROI result in different channel distributions. Although the inter-module channels are identical between modules, the resulting total number of channels is related to the number of modules needed to cover the ROI. The effect of module shape on channel distribution is complex and requires a tool such as MOCA to thoroughly investigate. Certain module geometries result in optodes closer to the module’s edges, effectively shortening inter-module channels in completely tessellated probes. Because the optode layout in Fig. 4 is not completely symmetric and each module shape is an equilateral polygon, each individual module can be re-oriented without overlapping while maintaining the complete tessellation of the probe. While not altering intra-module channel distributions, these orientation configurations spatially alter channel locations and alter inter-module channel separations. The results from Fig. 5 also show how some individual module shapes may be more appropriate for certain subject populations. For example, the high count of 19-mm inter-module channel separations in the hexagon-based probe make it better suited for infant populations. An important takeaway is that the number of inter-module channels of an assembled probe is not a simple multiplicative factor of the number of intra-module channels. These results demonstrate the dependency a probe’s derived characteristics have on module shape even when different modules have the exact same optode layout.

The results in Fig. 6 provide a counter-example where higher channel density due to increased inter-module channels may not necessarily improve all performance metrics of a probe. Despite having fewer total channels than the hexagon-based probe, the square-based probe results in a higher average brain sensitivity ($\overset{¯}{S_{\text{brain}}}$) due to larger inter-module channel separations. This reveals the trade-offs in performance metric improvement, emphasizing the need for $S_{\text{brain}}$ to be considered in conjunction with channel distribution when comparing probes. Additionally, this analysis reveals that the use of inter-module channels in addition to intra-module channels does not always lead to increased $S_{\text{brain}}$ for probes based on different module shapes. In fact, the use of only inter-module channels increases the average penetration depth for the square- and triangle-based probes due to the larger channel separations. However, for the hexagon-based probe design in this example, Fig. 6 shows that the contribution to $\overset{¯}{S_{\text{brain}}}$ from using only intra- or only inter-module channels differed by merely 0.1%. These results show that adding inter-module channels to intra-module probes will not always result in improved $\overset{¯}{S_{\text{brain}}}$. Thus, users of this particular hexagon-based probe may benefit from the simplicity and faster sampling rate of using only intra-module channels rather than implementing a potentially complex data acquisition method to capture inter-module channels. Although ignoring inter-module channels can increase sampling rate without affecting $S_{\text{brain}}$ for this particular probe, it does result in fewer channels and lowers the channel density of the probe. Through this complex example, we show that it is non-trivial to consider all constraints in a modular probe. MOCA is positioned as a tool to help designers challenge hypotheses, explore alternative designs, and quantify various trade-offs.

Figure 7 shows that the hexagon-based probe can achieve the highest sampling rate among the three configurations if a spatial multiplexing encoding strategy is implemented. The frame rate of a sequential encoding strategy is dependent on the total number of sources ($n_{s}$) because each source needs to be turned on and sampled once. Spatial multiplexing allows multiple sources within a group to be turned on simultaneously, allowing the sampling rate to increase by a factor of $n_{s}/n_{\mathrm{SMG}}$, defined as the SMR. Therefore, despite having the lowest sampling rate when sampled sequentially due to the highest $n_{s}$ (Table 2, Row 1), the hexagon-based probe has the fastest sampling rate of the three probes when spatial multiplexing is used due to the low $n_{\mathrm{SMG}}$ [Fig. 7(a)]. These results demonstrate that a probe’s sampling rate can be increased not only through firmware changes or advanced electronics, but also by using different module shapes with the same optode layout.

While MOCA’s ability to change module-level parameters helps design new fNIRS modules, its ability to sweep through probe-level parameters helps potentially improve existing ones. Figure 8 shows how probes based on published modules can potentially improve $\overset{¯}{S_{\text{brain}}}$ at no increased cost and without re-designing modules by altering the orientations of modules that make up the probe. The orientation changes in layout 66 [Fig. 8(c)] increase the channel density at the center of the ROI, but also increase the number of inter-module channels by 43%. The emerging inter-module channels also have larger source–detector separation (SDS) and contribute to an increase in $\overset{¯}{S_{\text{brain}}}$ without changing the SMR. The re-oriented probe in Fig. 8(c) is only a representative case of how a 2×2 probe composed of square modules can be potentially improved and is exhaustive only because the number of possible orientations of each of the four modules were limited to 4, resulting in $4^{4}=256$ probe layouts to analyze. Additionally, the re-orientation of modules causes changes to performance metrics due to the asymmetry of the optode layout within each module. If the optode layout was symmetric, re-orienting modules would have no effect on either inter- or intra-module channels.

In Fig. 9, we investigated the effect of spacing between modules on the derived performance metrics of a probe composed of hexagonal-shaped modules. The results suggest that varying module spacing does have an impact on $\overset{¯}{S_{\text{brain}}}$. Since optodes are generally placed near the edges of the modules to maximize intra-module channel separations, dense probes with modules near one another tend to have shorter inter-module channel separations. This trend becomes more apparent as the size of the module increases. Increasing the probe spacing increases the distance between optodes on neighboring modules, thus increasing the $\overset{¯}{S_{\text{brain}}}$ in the process. This increase in $\overset{¯}{S_{\text{brain}}}$, however, has a local maximum. As shown in Fig. 9, further increasing probe spacing leads to a drop in the number of inter-module channels as their SD separations become greater than the separation limit (${\mathrm{SDS}}_{\max}$). Additionally, increasing the distance between modules reduces the number of multiplexing groups ($n_{\mathrm{SMG}}$) which increases the SMR and consequently the probe’s sampling rate. Once the probe spacing exceeds the user-specified SMG diameter, one source on each module can be turned on at the same time because each source would be outside the other’s “effective” region. Because the distance between sources on the same module does not change, a different SMG is required for each source within a module. Thus, the limit to the minimum $n_{\mathrm{SMG}}$ is equal to the number of sources on a single module, revealing a minimum improvement in sampling rate due to spatial multiplexing encoding. Compared to a sequential sampling strategy, a spatial multiplexing encoding strategy will increase a probe’s sampling rate by at least a factor equal to the number of sources on one module. Figure 9 shows that probe spacing can both alter $n_{\mathrm{SMG}}$ to help meet sampling rate requirements and alter inter-module channel separations to meet channel distribution needs.

Figure 10 shows that staggering a probe layout can increase $\overset{¯}{S_{\text{brain}}}$ in dense probes. Simulations using a published module shape^30^ with zero probe spacing results in inter-module channels of 14-mm separations. These channels are too long to be SS channels and too short to be LS channels. Staggering spatially increases inter-module channel separations while maintaining the compactness of a probe [Fig. 10(c)]. This improvement works with square or rectangular modules since staggering is done by translating user-specified modules along a horizontal or vertical axis. For module designs with symmetrical optode layouts, we recommend staggering probe layouts by translating every other module row by half of the module’s maximum width in one axis [Fig. 10(f)]. This ensures the optodes from the translated module are well separated from modules of the adjacent rows.

The results above are derived from investigating the module- and probe-level design parameters that MOCA currently supports. However, this only represents a small subset of the general parameters previously used in evaluating a modular probe.^24^ For example, user feedback-based design parameters not yet accounted for in MOCA include conformability (a module’s ability to conform to a curved surface), subject comfort, and safety limits such as operating voltage and heating effects. Source output power and module weight each require external instrumentation measurements while noise-equivalent power and dynamic range calculations require lab-specific phantoms. Power consumption depends on the type of optodes used as well as the control electronics of the individual module while a probe’s battery life can be adjusted using existing off-the-shelf components. Each of these design parameters are based on specific electronic or material components chosen for a particular module design. MOCA was built to easily scale and incorporate more complex mechanical-, ergonomic-, safety- and experiment-specific considerations in the future as those design parameters are evaluated.

There are limitations to MOCA’s current minimal subset of design parameters. First, the ability to re-orient, increase spacing, or stagger modules assumes that modules can be connected in any orientation. This is true for many published modular designs where cables of different lengths can be easily connected to the top of a module, but does not necessarily apply to more sophisticated designs that have embedded printed flex connectors or utilize headgears with pre-determined mounting locations. Second, MOCA does not currently support multiple module shapes within the same probe or different optode layouts on different modules. Third, MOCA’s channel count output does not include wavelength number as a multiplier. This approach allows one to quickly scale the channel distribution and channel counts when dual-wavelength or triple-wavelength sources are utilized. Similarly, the $n_{\mathrm{SMG}}$ is also defined for a single wavelength. Thus, when estimating the total sampling rate of the probe using multi-wavelength sources, the control unit must cycle through each group multiple times (once for each wavelength). Fourth, MOCA’s analysis is based on the coordinates of the center of an optode’s active area and does not account for the actual size of optode package, the shape of the optode’s active area, or any master control unit needed to control a series of modules. Despite being able to place optodes near the edge of modules in MOCA, designers may face constraints in practice imposed by the fabrication process due to board materials, sizes, and electrical routing needed to drive these optoelectronics. In general, module shapes with large interior angles allow optodes to be placed closer to the module’s perimeter. Fifth, MOCA’s probe-level functions are only one way of interrogating parameters in a systematic way. These functions vary parameters in discrete increments (fixed number of orientations, a set spacing range, and user-defined translation amounts) and therefore only explore a subset of all potential probe layouts. They do not determine an optimal probe configuration—they assist in improving existing probes by adding design constraints (holding module-level parameters constant) and allowing a user to identify design choices that improve performance for their particular application. Finally, MOCA can output 2D optode layouts but relies on other existing software, such as AtlasViewer,^41^ to perform 3D head contour registration. It does, however, automatically add spring relationships to embed modular aspects into an exported probe. For example, the distance between all optodes (both sources and detectors) within a module are fixed, while inter-module channels can vary slightly. This ensures that a physically rigid electronic module does not transform when registered to a surface. In addition, the performance metrics output by MOCA are currently based on a 2D probe layout and do not account for changes in SDS if the probe is wrapped on the 3D surface of a head.^34^ Consequently, 2D derived metrics may underestimate the number of channels when a probe is made to conform to the scalp. This may result in an increase in the number of total inter-module channels for a probe. However, by working in 2D, MOCA can both help unearth design and performance relationships, as well as drastically constrain the vast potential design space by helping researchers converge on the module- and probe-level parameters that most impact performance.

## 6 Conclusion

We have developed a MATLAB-based modular probe design toolbox, MOCA, with the goal of providing fNIRS developers with a systematic yet easy-to-use software platform to navigate the large design space of modular fNIRS probes and provide metrics-based guidance. MOCA simplifies the design problem with module-level parameters, such as size, shape, and optode layout as well as probe-level parameters, such as the maximum SDS and ROI geometry to characterize a modular probe. It offers the ability to perform operator-guided sweeping of probe parameters, such as orientation, spacing, and module staggering offset, helping designers explore alternative designs that potentially improve upon existing probes or outline spectra of trade-offs. MOCA is quantitative, guided by application-specific fNIRS performance metrics, including channel distribution, average brain sensitivity, and spatial multiplexing groups, making it possible for quantitative characterization and comparison between various design decisions. Applying MOCA in several case studies, we identified several valuable design considerations that have not been widely recognized, including the importance of fine-tuning module orientation, spacing, and staggering distance. In the meantime, these case studies also demonstrate the complexity of modular probe optimization, where multiple variables compete and eventually lead to alternative designs with various trade-offs. While MOCA was not designed to provide full automation for complex probe design and optimization, it offers fNIRS probe designers a suite of powerful tools, including module tiling, routing, re-orientation, and fine-tuning of module spacing and staggering offset, with each outcome quantified by meaningful performance metrics. MOCA is expected to attract more research interests toward developing next-generation modular fNIRS systems.

## Code, Data, and Materials Availability

Our Modular Optode Configuration Analyzer (MOCA) toolbox is freely available for download at http://github.com/COTILab/MOCA.
